# Comparison of joint kinematics between upright front squat exercise and horizontal squat exercise performed on a short arm human centrifugation

**DOI:** 10.14814/phy2.16034

**Published:** 2024-07-01

**Authors:** Riccardo G. Sorrentino, Edwin Avila‐Mirèles, Jan Babič, Matej Supej, Igor B. Mekjavic, Adam C. McDonnell

**Affiliations:** ^1^ Department of Automatics, Biocybernetics and Robotics Jožef Stefan Institute Ljubljana Slovenia; ^2^ Jožef Stefan International Postgraduate School Ljubljana Slovenia; ^3^ Universal Robots, Research and Development Department Odense Denmark; ^4^ Faculty of Sport University of Ljubljana Ljubljana Slovenia

**Keywords:** artificial gravity, human centrifugation, motion analysis, movement adaptation, squat kinematic

## Abstract

This study compared the joint kinematics between the front squat (FS) conducted in the upright (natural gravity) position and in the supine position on a short arm human centrifuge (SAHC). Male participants (*N* = 12) with no prior experience exercising on a centrifuge completed a FS in the upright position before (PRE) and after (POST) a FS exercise conducted on the SAHC while exposed to artificial gravity (AG). Participants completed, in randomized order, three sets of six repetitions with a load equal to body weight or 1.25 × body weight for upright squats, and 1 g and 1.25 g at the center of gravity (COG) for AG. During the terrestrial squats, the load was applied with a barbell. Knee (left/right) and hip (left/right) flexion angles were recorded with a set of inertial measurement units. AG decreased the maximum flexion angle (MAX) of knees and hips as well as the range of motion (ROM), both at 1 and 1.25 g. Minor adaptation was observed between the first and the last repetition performed in AG. AG affects the ability to FS in naïve participants by reducing MAX, MIN and ROM of the knees and hip.

## 1 INTRODUCTION

Astronauts participate in tailored exercise programs before, during and after spaceflight (Korth, 2015; Loehr et al., 2015). Prior to missions, the exercise programme maintains their physical fitness, whereas during spaceflight the aim of the daily exercise programme is to mitigate the adaptation of the musculoskeletal and cardiovascular systems to weightlessness. Since exercise as a countermeasure to mitigate the deconditioning that occurs during longer sojourns in weightlessness is only partially effective, exercise is also part of the astronauts' rehabilitation programme upon return to Earth. The implementation of exercise countermeasures on the International Space Station (ISS) has reduced, but not eliminated the loss of muscle and bone mass (Stavnichuk et al., 2020). The efficacy of the current exercise strategy on the ISS may not be optimal for deep space missions, and thus new concepts are being investigated. During future Mars and Moon missions, astronauts will be exposed to less than 0.5 g for 493 and 180 days, respectively (Winnard et al., 2019). Implementing a strategy that could combine exercise with artificial gravity (AG) established with a short arm human centrifuge (SAHC) in future space habitats might provide an efficient countermeasure against microgravity‐induced adaptations.

The possibility of creating and utilizing AG, an idea proposed by Herman Potočnik Noordung (1929), is being assessed as a potential countermeasure. Whereas Noordung (1929) proposed a rotating space station that would establish Earth's gravity at the perimeter of the circular station, the current strategy is to use SAHC with radii varying from 2 to 5 m, to expose astronauts to AG for short durations daily (Clément, 2011; Clément, Charles, et al., 2016; Clément, Paloski, et al., 2016; Frett et al., 2014). Three strategies incorporating AG are currently being investigated by the European Space Agency (ESA). The first entails passive exposure to AG, and the remaining two incorporate either aerobic (Yang et al., 2010) or strength (Yang et al., 2007) exercise during exposure to AG. The unique feature of conducting exercise on a SAHC, is that it allows targeting all physiological systems during one exposure (Goswami et al., 2015; Laing et al., 2020; Yang et al., 2010), instead of relying on several exercise devices. Centrifugation per se initiates cardiovascular responses akin to those observed in the upright position (Goswami et al., 2015; Verma et al., 2018). During squat exercise on the SAHC, the radial acceleration vector acting on the body increases linearly from the axis of rotation, so that a participant lying supine on a SAHC with their feet distal to the central axis, will experience more force at the feet than on the upper body. Furthermore, a squat on the SAHC compared to an upright squat may be biomechanically different, resulting in a different pattern of muscle activation. Yang et al. (2007) have reported that it is possible to generate high ground reaction forces (GRF) during a squat performed on a centrifuge compared to the GRF generated during upright exercise. This study demonstrated that both technique and muscular activation are similar between the two conditions. However, this study used a human‐powered centrifuge, which is markedly different from motor‐powered centrifuges, particularly SAHC. A more recent study by Duda et al. (2012) investigated squat biomechanics performed on a SAHC and reported that centrifugation increases mediolateral knee travel (MLKT), but confirmed that an exercise protocol can be completed. Piotrowski et al. (2018) compared the metabolic cost of upright squats and squats performed on a SAHC and reported that squats performed on the SAHC had a lower oxygen uptake, which they attributed to diminished activation of trunk muscles as participants were positioned supine on the sled, thereby avoiding the need for torso stabilization.

The SAHC utilized in these and numerous prior investigations employed a single‐axis sled configuration, enabling movement solely along the frontal plane. Recently developed iterations of the SAHC feature an advanced 2‐axes sled system, distinguished by its additional rotational capability within the frontal plane. Such a system allows the engagement of hip movement (flexion/extension), which enables the squat on the SAHC to better mimic the conventional squat rather than a leg press. This feature holds significant implications for astronauts, as conventional leg press exercises and single‐axis sled systems typically restrict hip movement, resulting in isolation of leg muscles without engagement of the hip, gluteal and abdominal musculature. Consequently, crucial stabilizing muscles of the hip and abdomen, which play a pivotal role in maintaining proper posture and are subjected to significant adaptations in microgravity environments, remain underutilized. The recruitment of these muscles as part of the countermeasure exercise would therefore be beneficial (Qaisar et al., 2020; Vandenburgh et al., 1999).

For this reason, the aims of the present study were to: (i) assess the feasibility of conducting squat exercise on the SAHC with the novel 2‐axes sled system allowing flexion/extension of the hip joint, (ii) compare the kinematics of squat exercise performed on the SAHC with that of upright squat exercise, conducted at the same GRF, (iii) assess the magnitude of adaptation that occurs when performing squat exercise on the SAHC during one training session.

## 2 METHODS

**FIGURE 1:**
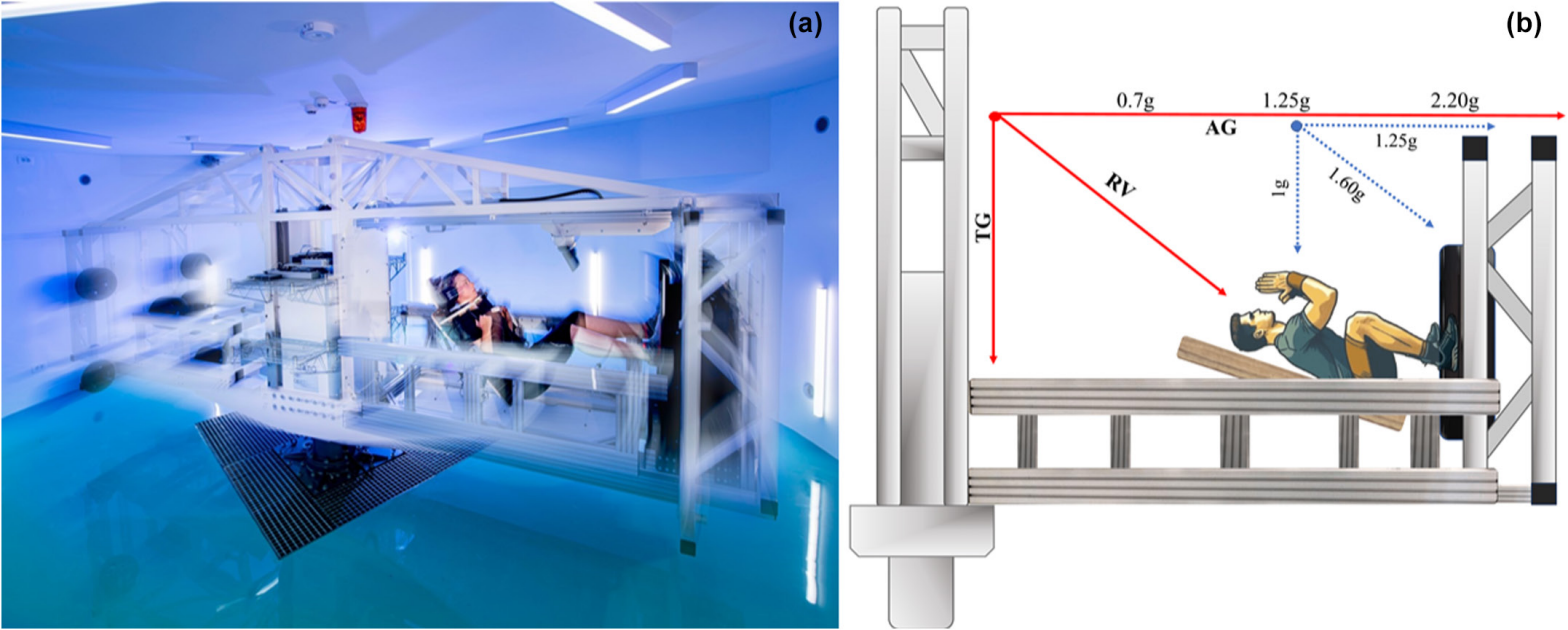
(a) European Space Agency (ESA) Short Arm Human Centrifuge (SAHC) in motion (photo credit: K. Bidovec and A. Hodalič). The SAHC has two nacelles. One for the participant and the other for the counterweights. The participant is positioned with the feet on a force platform, which can be activated to provide vibration. The unique feature of the SAHC is the sliding gurney on which the subject is positioned. It allows for squats to be performed, simulating an upright squat. Specifically, the swivel at the centre of mass allows hip flexion and extension during the squat exercise. (b) Establishing a ground reaction force of 2.2 g at the feet would result in a linear decrease in acceleration toward the head (see text for details). During the squat maneuver, the gravito‐inertial force on the subject will be the result of Earth's gravity and artificial gravity (AG).

The study was conducted at the ESA ground‐based facility PlanHab (Rateče‐Planica, Slovenia), which maintains ESA's SAHC (Figure 1). The SAHC (Qinetiq, Belgium, now acquired by Redwire, Antwerp, Belgium) has two nacelles, one for the participant, and another acting as a counterweight. The SAHC nacelle cradle comprises side mounting blocks affixed on a sliding rail and a cushioned back board that provides support from the coccyx to the head. The unique feature of this cradle is that it rotates around the mounting block allowing for movement in both the horizontal and vertical direction (AMST, Austria). For this reason, this system is referred to as the 2‐axis sled. The participant is positioned supine on the sled, and during centrifugation little input is required from the participant to maintain balance, however, when initiating a squat movement, the vertical displacement (up to 45°) allows the participants to flex their hips in a motion that would not be possible on a fixed sled. Figure 1 illustrates the manner in which the subjects were able to conduct horizontal front squat (FS) exercise on the SAHC, mimicking the squat exercise conducted in the upright position.

### 2.1 Participants

Healthy male subjects (*N* = 12) participated in the study. Their mean ± SD age was 22.7 ± 1.7 years, weight was 83.3 ± 6.1 kg, and height was 183.1 ± 6.1 cm. All participants met the inclusion criteria: age between 18 and 40, physical exercise at least thrice weekly, knowledge of how to accurately perform a squat, nonsmokers, height less than 195 cm and weight less than 95 kg. The latter two constraints were due to technical reasons associated with the SAHC. The study protocol was approved by the University of Ljubljana, Faculty of Sports' Committee for Ethical issues in the field of sport (Reference number: 033–10/2023–2). All participants provided their written informed consent to participate in the study, which was performed according to the guidelines of the Declaration of Helsinki, excluding clause 35 (i.e., the study was not registered in a publicly accessible database). A certified strength and conditioning coach gave final approval for participation of subjects in the study, once they were deemed capable of performing a FS according to the criteria of Kritz et al. (2009). Specifically, the participants had to be capable of executing the squat movement by reaching a peak knee flexion of 90° while maintaining neutral (upright) spine position. The participants wore sports clothing and footwear for this initial assessment and throughout testing.

### 2.2 Experimental protocol

The participants were requested to perform FSs in two exercise settings: (1) conventional upright squats (PRE and POST) and (2) squats on the SAHC under AG loading. The FS was the chosen exercise instead of a back squat as this may more accurately simulate the position maintained by the participants during squatting on the centrifuge. Based on the guidelines set out by Myer et al. (2014) and Yavuz et al. (2015) the back squat allows further thoracic lean than that noted in the FS variant. This reduced lean is similar to that noted in novice users of the centrifuge and therefore a kinematic comparison between the two exercise modalities may be valid.

In each setting, the participants performed three sets of six squats with 1 min of rest between sets under two different loads. The loads were either body weight (BW) or BW + 25%. In the AG setting these loads corresponded to 1 g or 1.25 g at the center of mass (COM) which is achieved by spinning the SAHC at an individualized speed based on the participants COM height and its distance from the central motor. BW and BW + 25% from heretofore will be referred to as 1 g and 1.25 g. Participants first conducted the exercise in an upright position (PRE), followed by squatting on the centrifuge (AG) and finally repeated the squats in an upright position after centrifugation (POST). There was a five‐min break between the exercise settings to allow the researchers to install the participant onto the centrifuge and remove them following AG. The conventional upright squat exercise was performed using a squat rack and barbell in a room adjacent to the SAHC, which minimized the transfer time between the exercise settings.

The order of exercise was always the same, however, the application of load was randomized in the PRE trials and then kept constant in the following conditions (AG and POST).

This protocol was chosen because optimal squats were required for the analysis to have proper squat kinematics and fatigue needed to be avoided. Fatigue was monitored with the Borg scale for subjective ratings of perceived exertion (RPE). The pace of 3 s per squat cycle (i.e., the down and up phase combined), was provided by using a metronome and vocal cues. Participants were given specific instructions and verbal feedback during upright exercises. This feedback related to maintaining proper chest posture and a neutral spine alignment while performing the FS. After completing PRE squats, the participant was secured to the centrifuge. The harness worn by the participant was secured to the centrifuge's frame to avoid the participant shifting toward the side of the sled during spinning. Once centrifugation was initiated, 1 min was required to reach the target acceleration in the head‐to‐foot direction, at which point the squat exercise commenced. During AG, participants were instructed to maintain a pace similar to that of upright exercise. Purposely, neither metronomes nor verbal cues were employed, as one of the study's objectives was to examine the performance of new users executing FS on the centrifuge and to discern potential movement adaptations. Verbal cues and instructions were only provided in instances when the observed squat maneuver was inappropriate. During the trials the participants rested for 2 min between sets. After completing the 1 g set on the SAHC (AG trial), the speed of the centrifuge speed was increased to simulate 1.25 g. This process took 1 min and after reaching the proper ground reaction force (GRF) of 1.25.

Upon completion of the AG trial, subjects repeated the same protocol in the upright position (POST trial).

The participants were equipped with a head‐set and microphone on the SAHC, which provided continuous communication with the lead researcher during centrifugation. The foot board and thus the participants' feet were 2.4 m from the center of rotation of the SAHC and were spaced ~45 cm apart.

### 2.3 Kinematics assessment

A wearable wireless motion capture system (MVN Awinda, Xsens, Enschede, Netherlands) was used to capture the participants movement during squatting. Data was sampled at 60 Hz. A full‐body configuration was used. Prior to the attachment of the sensors, prerecorded anatomical measurements obtained from each participant were registered in the software. These anatomical measurements were height, shoulder height and width, elbow dimensions, wrist dimensions, arm span, hip dimensions, as well as knee and ankle measurements. The placement, orientation, and positioning of sensors were established according to the guidelines specified by the manufacturer and validated in prior research studies (Schepers et al., 2018). Subsequently, each participant underwent sensor calibration. The calibration procedure involved maintaining an upright standing posture with relaxed arms extended straight alongside the trunk with palms of the hands parallel to the thighs, followed by walking forward, returning to the initial position, and maintaining the original posture. If the software deemed the calibration satisfactory, the participant proceeded with the experiment; otherwise, the calibration process was repeated until it was deemed acceptable. The participants' movement data for the knee and hip angles were exported for further analysis. Participants were instrumented with 15 sensors, which were secured with Velcro® straps on specific anatomical locations. Single sensors were placed on the sternum, at the back of the head and on the lower back. Paired sensors were strapped on the scapula, at the base of the deltoids, wrists, on the vastus lateralis, on the surface of the shins and feet.

The Xsens MVN Awinda Analyze system was used to record the participants' movement and to extrapolate knee and hip flexion/extension angles. Data was sampled at 60 Hz. A full body configuration was used.

### 2.4 Data analysis

Four joints were chosen for the analysis, that is, right/left hip and right/left knee. The minimum (MIN) and maximum flexion (MAX) and range of motion (ROM) that is, the range of joint movement from the maximum to the minimum flexion (MIN) point, were analyzed for both the participants' knee and hip joints during the squatting exercise. Initial analyses found no difference between the right and left selected joints and as such the data was combined and presented as simply knees and hips. MIN was defined as the angle when the participant was fully extended, and just prior to the onset of the down‐phase of the FS. MAX was defined as the angle when participants reached the bottom position of the FS, and just prior to the up‐phase of the FS. Data comparisons was performed between three conditions: FS before centrifugation (PRE), FS on the centrifuge (AG) and FS after centrifugation (POST). To investigate the MLKT, only the MAX was taken as value. Single joint data are available in the Supplementary data.

The first and the last repetition of the middle set were discarded and thereafter the average of the remaining repetitions (the middle set under each load with four repetitions per set) were taken into account. These combined average values were then utilized for the subsequent statistical analyses. The selection of repetitions as described ensures a good degree of data analysis reliability for kinematic investigations, in accordance with previous literature findings (Frykberg et al., 2021). Data comparisons were performed between three exercise settings (PRE, AG and POST). All data were tested for normality distribution with a Shapiro–Wilk test due to the sample size.

The kinematic characteristics of both TS and AG exercises and the impact of varying loads were compared with a two‐way repeated measures ANOVA. This analysis incorporated two factors: exercise (*conditions*: PRE, AG, POST) and load (*loads*: 1–1.25 g). Statistical significance for which the hypothesis would be accepted was set a priori as *p* < 0.05. Tukey's post hoc test was performed if a main effect was identified among the *condition x load* interaction and/or its singular components.

A two‐tailed paired t‐test was performed between the first and the last repetition performed on the centrifuge; for this analysis, only the first and the last repetition of the full centrifugation session were analyzed regardless of the load (1 g and 1.25 g). When an adaptation was observed, an additional 2‐way repeated measures ANOVA was performed specifically within the AG session. This analysis considered the variables of repetitions (*timepoint*: first and last), and load levels (*load*: 1–1.25 g), in order to examine the precise location at which the adaptation took place. An analysis of effect size was also conducted using Hedges' *G* test. Given the relatively small sample size in this study (less than 20), this approach was chosen to mitigate potential bias, with the incorporation of a correction factor (Sullivan & Feinn, 2012). The statistical analysis and visual data representation was performed with GraphPad Prism 9 (Dotmatics) and Hedges' G calculations with a Microsoft Excel add in.

## 3 RESULTS

All participants successfully completed all trials. During PRE and POST FS, participants performed the exercise correctly, meeting the guidelines of a well‐executed exercise (Kritz et al., 2009).

### 3.1 Knee kinematics

**FIGURE 2:**
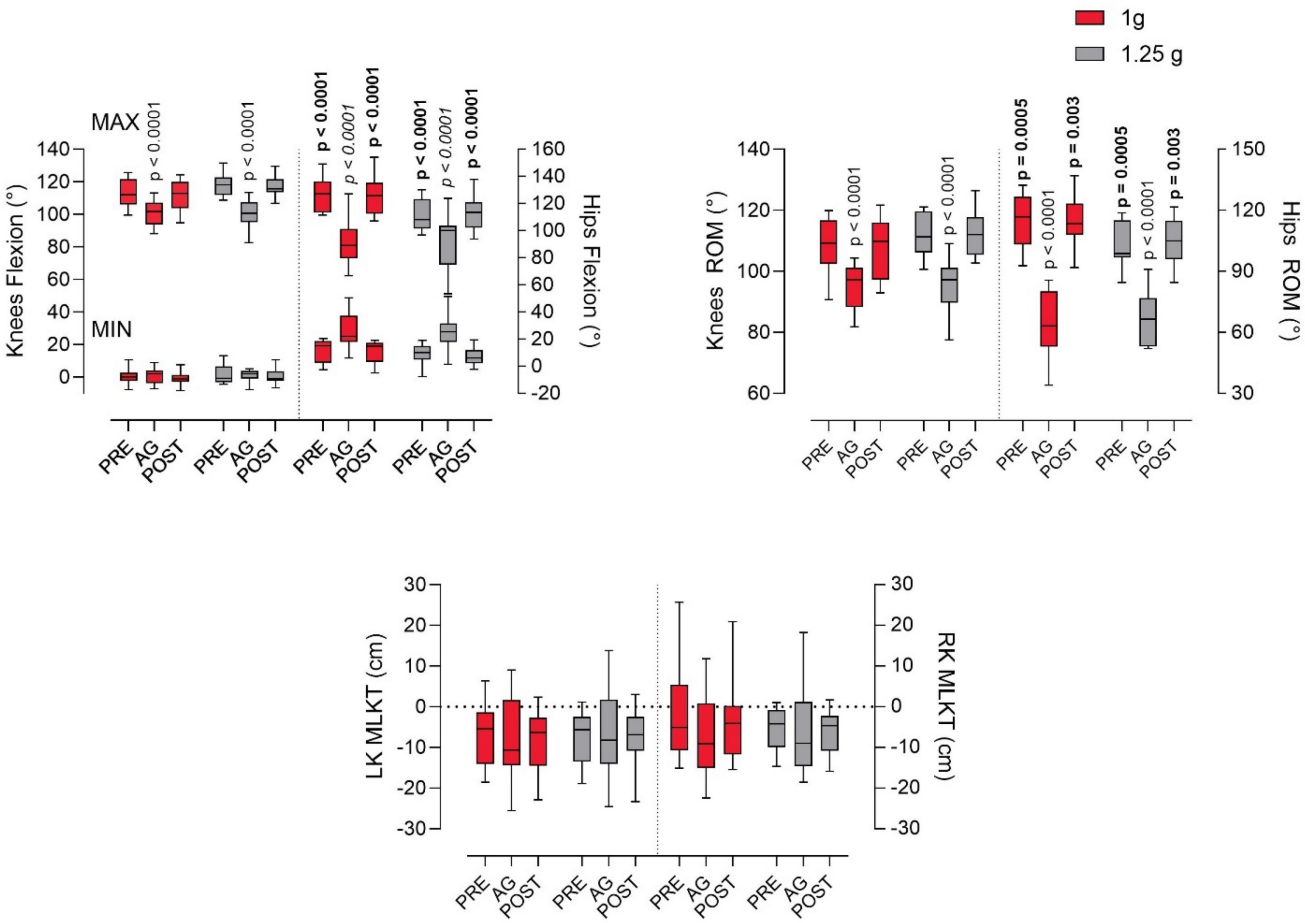
Minimum to maximum and mean values are represented in the box and whisker plots. Knees and hips flexion (top left panel) and range of motion (ROM, top right panel), and left (LK) and right knee (RK) medial lateral knee travel (MLKT) in trials conducted before (PRE), after (POST) and on the SAHC establishing a load at the center of mass (artificial gravity, AG) of 1.0 g and 1.25 g. For MLKT, a positive value signifies lateral knee deflection in the frontal plane (outward), whereas a negative value signifies a medial deflection (inward). Regular font = differences between AG, and PRE and POST; bold font = indicates difference between loads in the same condition (PRE 1 g vs. PRE 1.25 g, and POST 1 g vs. POST 1.25 g); italic font = difference between AG, and PRE and POST for MIN flexion.

No interaction of *load x condition* (F (2,22) = 1.002; *p* = 0.38) was observed; however, a significant source of variation was identified within the *condition* factor (F (2,22) = 39.59; *p* < 0.001). The multiple comparison test revealed a decrease of knee ROM by 13% during the exercise in AG with a GRF of 1 g, compared to both PRE (G = −1.60; *p* < 0.001) and POST (*G* = −1.46; *p* < 0.001) trials during which the subjects were exposed to terrestrial gravity (TG). A further reduction of 15% was observed in both PRE (*G* = −1.96; *p* < 0.001) and POST (*G* = −1.93; *p* < 0.001) exposure states at 1.25 g, when compared to the exercise performed at the same GFR (1.25 g) in the AG trial. This reduction in ROM is attributable to knees MAX flexion, given that for knees MIN flexion there were no statistically significant differences either for the main effect of *condition x load* (F (2,22) = 0.77; *p* = 0.47) or for its singular components (*condition* = F (2,22) = 0.33; *p* = 0.71; load = F (2,22) = 1; *p* = 0.33; *G* = n.s.). On the contrary, the model for knees MAX reported no source of variation on *condition x load* interaction (F (2,22) = 1.65; *p* = 0.21), but on its singular components (*condition* = F (2,22) =28.61; *p* < 0.0001; *load* = F (2,22) = 8.99; *p* = 0.01). The multiple comparison test revealed a decrease of knee MAX flexion by 12% in AG compared to both PRE (*G* = −1.50; *p* < 0.001) and POST (*G* = −1.21; *p* < 0.001) exposure at TG (1 g), and a further reduction of 16% in both PRE (*G* = −2.17; *p* < 0.001) and POST (*G* = −2.16; *p* < 0.001) at 1.25 g, when compared to the exercise conducted at the same GRF during the AG trial. All multiple comparisons are depicted in Figure 2 and Table 1.

**TABLE 1:** Kinematic data and multiple comparisons test results between conditions. All data are presented as: Mean ± Standard deviation.

	PRE	AG	POST	PRE vs AG (p‐value)	POST vs AG (p‐value)	PRE vs POST (p‐value)
Knee kinematics						
ROM (1 g)	108.62 ± 9.60	94.81 ± 7.51	108.06 ± 9.78	*p* < 0.0001	*p* < 0.0001	*p* = 0.96
ROM (1.25 g)	112.29 ± 7.11	94.96 ± 9.71	112.24 ± 7.32	*p* < 0.0001	*p* < 0.0001	*p* = 0.99
MAX flexion (1 g)	113.52 ± 8.44	100.43 ± 8.36	112.03 ± 9.95	*p* < 0.0001	*p* < 0.0001	*p* = 0.98
MAX flexion (1.25 g)	118.31 ± 6.99	100.36 ± 8.83	117.32 ± 6.02	*p* = 0.0001	*p* < 0.0001	*p* = 0.99
MIN flexion (1 g)	4.90 ± 4.64	5.62 ± 4.86	3.96 ± 3.71	*p* = 0.80	*p* = 0.20	*p* = 0.65
MIN flexion (1.25 g)	6.02 ± 5.77	5.39 ± 3.92	5.08 ± 4.89	*p* = 0.86	*p* = 0.98	*p* = 0.65
RK MLKT (1 g) *	‐1.72 ± 12.54	‐7.59 ± 9.89	‐3.89 ± 9.56	*p* = 0.26	*p* = 0.29	*p* = 0.33
RK MLKT (1.25 g) *	‐5.27 ± 5.35	‐6.55 ± 10.60	‐5.83 ± 5.26	*p* = 0.88	*p* = 0.96	*p* = 0.75
LK MLKT (1 g) *	‐6.15 ± 8.05	‐8.53 ± 10.58	‐8.07 ± 7.27	*p* = 0.06	*p* = 0.88	*p* = 0.95
LK MLKT (1.25 g) *	‐8.10 ± 6.71	‐7.48 ± 11.24	‐7.81 ± 7.23	*p* = 0.81	*p* = 0.94	*p* = 0.95
Hip kinematics						
ROM (1 g)	114.48 ± 12.91	65.43 ± 16.05	114.80 ± 13.41	*p* < 0.0001	*p* < 0.0001	*p* = 0.99
ROM (1.25 g)	102.42 ± 10.97	67.68 ± 12.99	104.98 ± 11.43	*p* < 0.0001	*p* < 0.0001	*p* = 0.67
MAX flexion (1 g)	126.11 ± 12.05	91.96 ± 18.35	125.63 ± 13.50	*p* < 0.0001	*p* < 0.0001	*p* = 0.98
MAX flexion (1.25 g)	111.40 ± 11.27	93.79 ± 20.95	112.16 ± 12.31	*p* < 0.0001	*p* < 0.0001	*p* = 0.95
MIN flexion (1 g)	11.62 ± 8.28	26.53 ± 12.86	10.82 ± 8.12	*p* < 0.0001	*p* < 0.0001	*p* = 0.89
MIN flexion (1.25 g)	8.97 ± 7.79	26.10 ± 14.15	7.17 ± 6.48	*p* < 0.0001	*p* < 0.0001	*p* = 0.59

*Note*: The asterisk (*) signifies that numbers are expressed as centimeters (cm) and not as degrees of flexion (°) as the other data.

### 3.2 Hip kinematics

A statistically significant *condition x load* interaction was observed for the hip movements during the squat exercise (F (2,22) = 6.65; p = 0.005) as well on its singular components (*condition* = F (2,22) = 86.77; *p* < 0.0001; *load* = F (2,22) = 15.28; *p* = 0.002). Tukey's multiple comparisons test revealed a significant ROM decrement in AG of 42% compared to PRE (*G* = −3.25; *p* < 0.001) and POST (*G* = −3.23; *p* < 0.001) at 1 g, and of 33% compared to PRE (*G* = −2.78; *p* < 0.001) and POST (*G* = −2.94; *p* < 0.001) at 1.25. Moreover, a ROM reduction of 10% occurred also between loads (1 g vs. 1.25 g) in PRE (*G* = 1; *p* = 0.006) and POST (*G* = 0.78: *p* = 0.03).

Unlike the knees, hip ROM reduction is caused by both a reduction of hips MAX (*condition x load* = F (2,22) = 6.65; p = 0.005) and an increase of hips MIN (*condition* = F (2,22) = 0.82; *p* = 0.45). Tukey's post hoc analysis revealed a 27% reduction of hips MAX in AG compared to PRE in 1 g (*G* = −2.12: *p* < 0.001) and POST (*G* = −2.01; *p* < 0.001) and of 15% compared to PRE (*G* = −1.01; *p* < 0.001) and POST (*G* = −1.03; *p* < 0.001) in 1.25 g. Hips MAX decreased of 13% between loads within the same condition (1 g vs. 1.25 g) both in PRE (*G* = 1.26; *p* = 0.003) and POST (*G* = 1.04: *p* = 0.009).

Hips MIN increased by 56% in AG compared to PRE (*G* = 1.33; *p* < 0.001) and POST (*G* = 1.41; *p* < 0.001) at 1 g, and by 65% at 1.25 g compared to PRE (*G* = 1.44; *p* < 0.001) and POST (*G* = 1.66; *p* < 0.001). No load effect was recorded for hips MIN.

### 3.3 MLKT

There was no main effect for either both knees (RK *condition x load* = F (2,22) = 0.19; *p* = 0.72; LK *condition x load* = F (2,22) = 2.41; *p* = 0.11), or for the singular components (*condition* and *load*). No differences were found between right and left knee during AG both in 1 and 1.25 g. Small and moderate effects were recorded for RK in 1 g for PRE (*G* = 0.51) and POST (*G* = 0.38).

### 3.4 Acute adaptation

**FIGURE 3:**
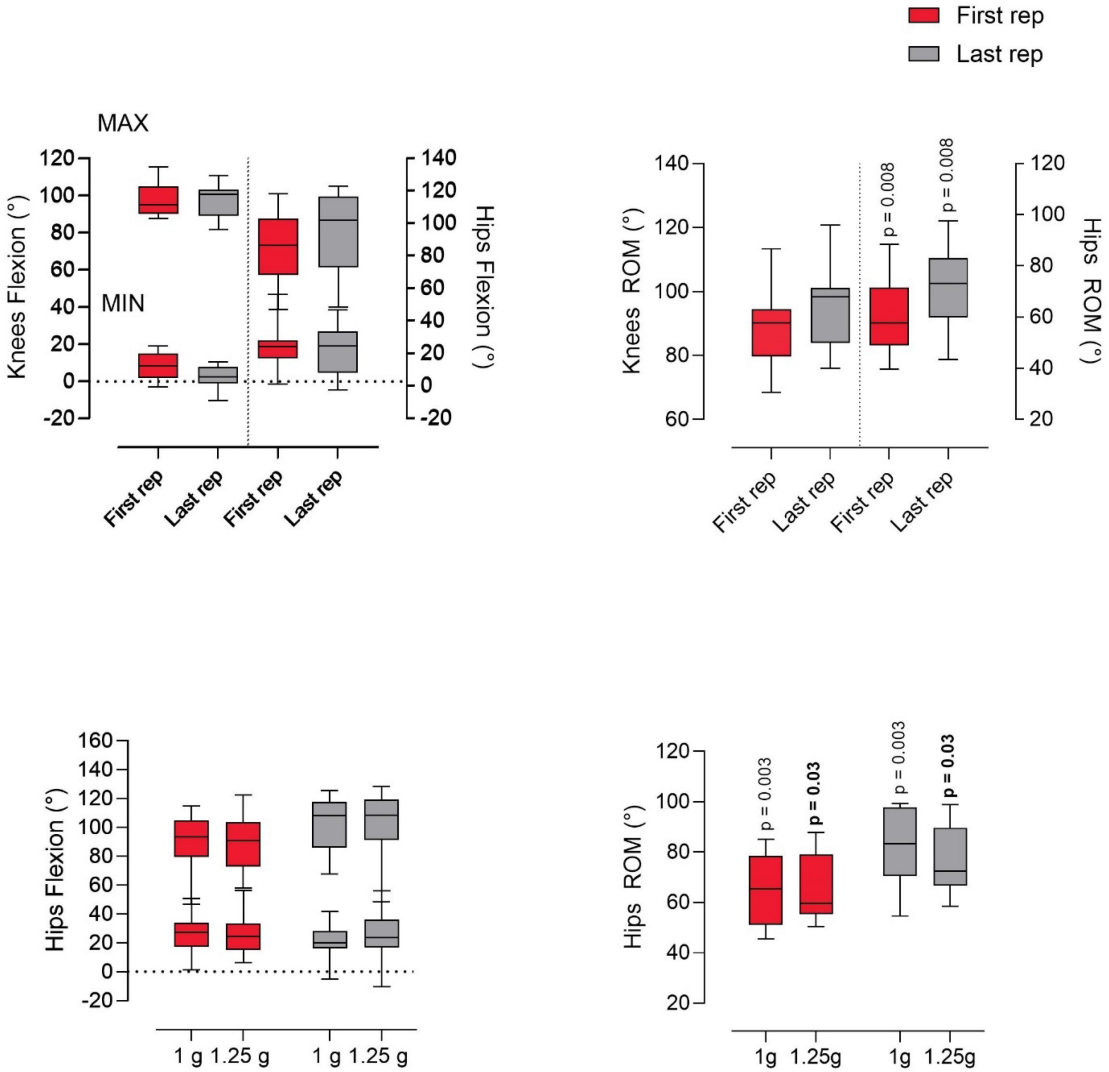
Minimum to maximum and mean values are represented in the box and whisker plots. Adaptation of knees and hips flexion (top left panel) and knees and hips range of motion (ROM, top right panel), as reflected in the difference between the first and last (rep) squat performed on the SAHC. Regular font = difference between the first and the last repetition. The lower panel presents the adaptation to the AG loads, specifically hips flexion (bottom left panel) and hips ROM (bottom right panel). Regular font = significant difference between first and last repetition at 1 g; bold font = significant differences between first and last repetition at 1.25 g.

No significant differences between the first and the last repetition were observed through post‐hoc analysis for knees ROM, MAX and MIN (Figure 3). However, moderate to large effect sizes were recorded for knees MIN (*G* = 0.86) and knees ROM (*G* = 0.48). No significant differences were found for hips MAX and MIN, while a small effect size (*G* = 0.44) was observed for hips MAX. Hips ROM was larger during the last repetition compared to the first one of 17% (*t* = 3.215, df = 11; *p* = 0.008).

With regards to hips acute adaptation, Tukey's post hoc analysis revealed no significant differences for MAX and MIN between first and last rep of both 1 and 1.25 g. A moderate effect (G = 0.58) was observed for hips MAX between the first and the last repetition in 1 g and a moderate effect (*G* = 0.50) for 1.25 g. A small effect was observed for hips MIN (*G* = 0.43) in 1 g. On the contrary, hips ROM was 21% larger in the last compared to the first rep at 1 g (*G* = 1.20; *p* = 0.003) and of 15% at 1.25 g (G = 0.84; *p* = 0.03). All data are depicted in Figure 3 and Table 2.

**TABLE 2:** Acute adaptation kinematic data, *t*‐test results and multiple comparisons results between conditions.

	First repetition	Last repetition	First vs last (p‐value)
Knees kinematics			
ROM	88.98 ± 11.69	94.83 ± 12.59	*p* = 0.20
MAX flexion	97.39 ± 8.59	97.62 ± 8.98	*p* = 0.93
MIN flexion	8.41 ± 6.97	2.78 ± 5.98	*p* = 0.057
Hip kinematics			
ROM	60.02 ± 14.34	72.76 ± 16.58	*p* = 0.008
MAX flexion	84.80 ± 20.41	95.10 ± 25.45	*p* = 0.09
MIN flexion	24.78 ± 13.37	22.34 ± 15.48	*p* = 0.56
ROM (1 g)	65.03 ± 13.67	82.35 ± 14.94	*p* = 0.003
ROM (1.25 g)	65.51 ± 13.21	77 ± 14.03	*p* = 0.03
MAX flexion (1 g)	90.72 ± 19.40	102.42 ± 20.31	*p* = 0.11
MAX flexion (1.25 g)	90.01 ± 20.71	101.10 ± 23.07	*p* = 0.12
MIN flexion (1 g)	25.68 ± 12.64	20.07 ± 13.34	*p* = 0.27
MIN flexion (1.25 g)	24.49 ± 13.93	24.09 ± 16.49	*p* = 0.93

*Note*: All data are presented as: mean ± standard deviation.

## 4 DISCUSSION

The principal finding of the present study is that participants naïve to exercise on a SAHC cannot replicate the kinematics of squat exercise performed in terrestrial settings (TS), when the exercise is performed during AG on the SAHC. Moreover, a mild adaptation process occurs already within a single centrifugation session.

### 4.1 Kinematic response to centrifugation

During traditional FS all participants conformed to the observed kinematics of a conventional squat movement, as documented in prior studies (Gullett et al., 2009; Kritz et al., 2009). The FS exercise was selected in this study, because it is a multi‐joint movement producing substantial internal forces, which has the potential to mitigate the muscle atrophy experienced by astronauts when conducted on a regular basis in reduced gravity environments (Escamilla, 2001; Escamilla et al., 1998). Compared to other well‐established lower limb exercises, such as the leg press, the squat elicits higher levels of muscular activity and places increased stress on the knee joint (Escamilla, 2001). Furthermore, the squat necessitates the engagement of hip stabilization muscles and the posterior chain to a greater extent compared to the leg press.

In the context of the study, when participants repeated squats in AG, they were unable to replicate the movement pattern they exhibited during upright squats. Specifically, the ROM in both the hips and knees was found to be significantly reduced in AG. Upon further analysis of the ROM, which comprises MAX and MIN, it was observed that the reduction in knee ROM occurred primarily at MAX flexion, while at MIN flexion, participants were able to fully extend their knees to the starting position with no discernible differences. The load did not have any effect on participants' response to centrifugation, since no differences were recorded between AG at 1 and 1.25 g.

In contrast, the reduction in hip ROM in the altered gravitational environment (AG) was attributed to a combination of decreased MAX and increased MIN. This reduction was the result of a failure to achieve complete flexion of hips as in TS and an inability to achieve full extension of the hips. Consequently, participants were unable to return their hips to a neutral position, resulting in each repetition starting from a partially flexed hip position. This adaptation can be explained by differences in the gravitational vectors exerting influence on the body during TS and in AG. In the context of TS exercises, whether loaded or unloaded, the sole acceleration vector impacting the participating subject is the unchanging force of Earth's gravity with or without the additional load.

Conversely, as described by Clement (2011) and co‐workers (2016), multiple acceleration vectors are present during centrifugation (see Figure 1b) on the SAHC: (i) natural gravitational vector, (ii) AG vector generated by the centrifugal force during the centrifugation, and (iii) gravito‐inertial force (GRIF) vector, which is the result of the interaction between the first and second vectors. The magnitude of this vector surpasses that of both vertical (natural gravitational vector) and horizontal vectors (AG). The example in Figure 1b presents a scenario of a participant lying supine on the SAHC with a rotational speed generating 1.25 g at the Center of Gravity (COG), and 2.2 g at the feet. The gravito‐inertial force in this example amounts to 1.60 g. The g_z_ values presented on the red arrow denote the gravitational load exerted on the participant in the supine position. During the down phase of the squat maneuver, the COG will move toward the feet, causing the artificial gravitational forces at the head and COG to increase. Thus, the dynamic interaction is not confined to a singular reference point; rather, at any point on the body, participants encounter a range of gravito‐inertial forces throughout AG exercise. During the squat manoevre, the gravito‐inertial force on the subject (resultant vector, [RV]) will be the result of the TG and AG. The RV generates a force that exerts a gravitational pull on the hips, a phenomenon distinctive to our experimental arrangement. Consequently, participants require multiple sessions to acquire the skill of activating their gluteal muscles in order to transition from a squatting position to a fully upright posture. The sled used in this study is similar to a system validated in previous research conducted under terrestrial conditions (Kramer et al., 2012, 2017) and more recently within a centrifuge setting (Kramer et al., 2020). Such findings support the notion that this two‐axis slide system enables the execution of jumping exercise, which involves the engagement of muscles activated during hip movement. It is important to note that an exact replication of the same movement could not be achieved, and kinematic distinctions were observed between exercises performed in the centrifuge and in the ground counterpart. The significance of utilizing a sled apparatus lies in its potential to engage a broader array of muscles during a single exercise.

The differences observed between TS and AG squats can be attributed to variations in the loads encountered by the participants. In TS, the load remains consistent throughout the entire ROM, meaning that the load applied to the barbell during the squat remains constant and it is perceived by participants as such, during both the eccentric and concentric phases of the exercise. In contrast, during AG, there is a linear increase in acceleration along the length of the nacelle, minimal near the central rotor and maximal at the end of the nacelle. When a subject lies supine on the nacelle on the SAHC, the acceleration along the length of their body also increases linearly toward the feet.

During the descent phase of the squat exercise on the SAHC, participants encounter an increasing load sensation due to the increasing head‐to‐foot artificial gravitational force. Conversely, during the ascent (concentric) phase of the squat, the load gradually diminishes, resulting in an actual and perceived reduced weight. In conventional upright squatting, biomechanical outcomes are influenced by variables such as the positioning of the barbell (high or low), the type of squat (front or back squat), the stance technique (wide or narrow) and the load. Therefore, the distinctly contrasting mechanics of load generation and perception during the ascending and descending phases of AG squats may play a contributory role in the modification of knee and hip kinematics when compared to those observed during TS. Given the low load of the exercise, it is unlikely that fatigue had an effect on the observed differences. As mentioned above, all participants were experienced in gym training, and the protocol was designed to minimize fatigue while providing a stimulus greater than body weight alone. Moreover, the rest intervals between sets and conditions were substantially longer than those typically recommended for resistance training with heavier loads. The highest reported rating of perceived exertion following an exercise, obtained with the Borg scale, was 12, indicating that the participants perceived the exercise to be light.

Our findings support those previously observed by Duda et al. (2012), that of an adaptation to centrifugation in a single AG set. Our data indicate that this adaptation extends beyond hip kinematics to include knee kinematics as well. Although no statistically significant differences were detected in knee kinematics, the effect sizes suggest an ongoing process in the knees as well. It is possible that an additional set may have made these differences more evident.

### 4.2 Load effect

There was no effect of load on the kinematic outcomes for flexion‐extension of knees (MAX, MIN) and ROM between all conditions. In contrast, load influenced hip ROM and MAX flexion of AG PRE/POST squats, but not the AG squats.

Hip ROM and MAX flexion differences between PRE and POST squat loads can be explained by the increased load applied on the barbell. As reviewed by Schoenfeld (2010), elevating the load within the range of 40%–70% of the one‐repetition maximum (1RM) prompts a greater degree of forward trunk inclination, and individuals with compromised hip mobility may exhibit diminished hip flexion mechanics. In the case of the FS, where the load is positioned on the chest, this phenomenon becomes more pronounced in comparison to the back squat. Even loads below those recommended by Schoenfeld may accentuate restricted hip movement, given the heightened demand for flexibility in the posterior kinetic chain during FS. It is worth noting that our study utilized only two loads. Future investigations should consider employing various levels of loading to assess whether differences emerge within AG at varying degrees of loads (i.e., 1.50 g, 1.75 g, etc.).

### 4.3 MLKT

This study investigated whether AG causes an increase in MLKT. Data were extrapolated within the same repetitions employed to investigate knee and hip movement. As pointed out by Duda et al. (2012) the knees internal deflection during the MLKT produces a torque, which can damage the knee joint if repeated over a longer term. Continuous internal rotation or unwanted abduction of the knees can lead to long‐term joint pathologies and the optimal kinematics would be to maintain a neutral position during squatting or jumping exercise (Han et al., 2013). The internal deflection of the knee is a problem during exercises involving lower limbs (Duda et al., 2012; Kramer et al., 2020; Piotrowski et al., 2018), especially during centrifugation. One of the main problems during centrifugation is the Coriolis effect, which can exaggerate medial or lateral deflection of the knees during both the eccentric and concentric phases. In contrast to the findings of Duda et al. (2012), this study did not report any statistically significant differences in MLKT between conditions. Furthermore, no differences were observed between the right and left knee in AG. A moderate and a small effect size between PRE/POST and AG were observed for RK only in 1 g. The disparities in findings between the current study and that of Duda et al. (2012) may be attributed to the differential cadence at which participants were instructed to perform squats. In the study of Duda et al. (2012), participants executed squats at a cadence of 2 s per repetition during upright and centrifugation squats. In the present study, participants were directed to adhere, as closely as possible, to the pace employed during the PRE and POST squats, which involved a descent and ascent phase of 3 s. During AG squats, participants were allowed to perform squats at their preferred pace, resulting in a longer duration for each repetition of up to 6 s per repetition. The faster squatting pace in the study of Duda et al. (2012) may explain why they observed an increased MLKT during AG squats, as higher speeds amplify the Coriolis effect. The slower execution speed of squats in the current study could have contributed to enhanced control over knee positioning in relation to abduction/adduction movements during both descent and ascent phases, potentially preventing an escalation in MLKT when compared to PRE and POST FS and decreasing the momentum.

### 4.4 Muscle activation

The goal of any exercise countermeasure is to induce sufficient activation of target skeletal muscles to mitigate the loss of muscle mass and function during space missions. The muscles of the thighs and calves are most affected by the inactivity/unloading experienced by astronauts on the ISS (Berg et al., 2007; Clément, 2011), and by people involved in simulated microgravity environments (Berg et al., 2007; McDonnell et al., 2019; Pišot et al., 2008) thus, any exercise strategy should focus on these muscles. The difference in the kinematics observed between the PRE and AG conditions would suggest that SAHC with a 2‐axes sled allows the performance of squat exercise safely, nevertheless, participants without any familiarization trials cannot recreate their upright FS movement. This could result in a different pattern of muscle activation in AG compared to the PRE and POST trials. Although, no data regarding muscle activation of the muscles involved in the exercise were recorded in this study. We suggest, in line with the findings that we have from upright typical squat and leg press exercise, that the squat, especially the front variant, is a superior exercise to elicit higher forces on lower limbs (Larsen et al., 2022; Yavuz et al., 2015). Furthermore, the squat necessitates the engagement of hip stabilization muscles and the posterior chain to a greater extent compared to the leg press (Schoenfeld, 2010). When coupled with the utilization of a SAHC featuring a 2‐axes system, the effect of a squat exercise on muscle activation may surpass those achievable with centrifuges employing only a single‐axis system. Moreover, the gravito‐inertial vector acting on the participant during AG squats most likely generates different torques on the joints between the two types of exercises and thus may result in differences in muscle activation.

### 4.5 Limitations and future perspectives

This is the first study monitoring the FS exercise kinematics on a novel SAHC sled. In the present study, EMG data were not obtained, as the primary emphasis was on kinematics. Future investigations should contemplate the inclusion of EMG measurements during SAHC squats and their comparison with upright exercises to explore potential disparities in muscular activation and quantify such differences.

## 5 CONCLUSION

The FS exercise conducted during AG on the SAHC is a promising countermeasure. The two‐axis sled system allows a FS to be performed that mimics to some extent the FS movement performed in the upright position. The influence of the interaction of the two acceleration vectors, however, affects the participants' ability to perform exactly the same movement as in the upright FS in natural gravity, especially the flexion‐extension of the hips. Naïve participants demonstrated some, albeit not significant, acute adaptation to SAHC, which might be more evident after several sessions of training.

## FUNDING INFORMATION

This study was supported, in part, by the Slovenian Research and Innovation Agency (ARiS Programme grant no. P2‐0076).

## Data Availability

The raw data derived from this study will be made available by the authors, without undue reservation.
